# Xenotransplantation of Embryonic Pig Kidney or Pancreas to Replace the Function of Mature Organs

**DOI:** 10.1155/2011/501749

**Published:** 2010-12-28

**Authors:** Marc R. Hammerman

**Affiliations:** Renal Division, Departments of Medicine and Cell Biology and Physiology, George M. O'Brien Center for Kidney Disease Research, Washington University School of Medicine P.O. Box 8126, St. Louis, MO 63110, USA

## Abstract

Lack of donor availability limits the number of human donor organs. The need for host immunosuppression complicates transplantation procedures. Ultrastructurally precise kidneys differentiate in situ following xenotransplantation in mesentery of embryonic pig renal primordia. The developing organ attracts its blood supply from the host, obviating humoral rejection. Engraftment of pig renal primordia transplanted directly into rats requires host immune suppression. However, insulin-producing cells originating from embryonic pig pancreas obtained very early following initiation of organogenesis [embryonic day 28 (E28)] engraft long term in nonimmune-suppressed diabetic rats or rhesus macaques. Engraftment of morphologically similar cells originating from adult porcine islets of Langerhans (islets) occurs in rats previously transplanted with E28 pig pancreatic primordia. Here, we review recent findings germane to xenotransplantation of pig renal or pancreatic primordia as a novel organ replacement strategy.

## 1. Introduction

Transplantation of embryonic renal or pancreatic primordia to replace the function of diseased organs offers theoretical advantages relative to transplantation of either pluripotent ES cells or of fully differentiated (adult) organs (Reviewed in [1, 2]). (1) Unlike embryonic stem (ES) cells, organ primordia differentiate along defined organ-committed lines. There is no requirement to steer differentiation and no risk of teratoma formation; (2) the growth potential of cells within embryonic organs is enhanced relative to those in terminally differentiated organs; (3) the cellular immune response to transplanted primordia obtained early during embryogenesis is attenuated relative to that directed against adult organs; (4) early organ primordia are avascular. The ability of cellular primordia to attract a host vasculature renders them less susceptible to humoral rejection than are adult organs with donor blood vessels transplanted across a discordant xenogeneic barrier; (5) organ primordia differentiate selectively. In the case of embryonic pancreas, exocrine pancreatic tissue does not differentiate following transplantation, obviating complications that can result from exocrine components such as the enzymatic autodigestion of host tissues. 

While the transplantation of human embryonic organs in human hosts has been contemplated [3–5], we [6–13] and others [4, 5, 14–17] have focused on the use of embryonic organs from the pig, a physiologically suitable donor for human pancreas or kidney replacement [18, 19].

## 2. Xenotransplantation of Embryonic Pig Kidney

Pig renal primordia are “preprogrammed” to differentiate into a kidney after transplantation into the mesentery of hosts with reduced functional renal mass (unilateral nephrectomy). Differentiation without immune rejection occurs following allotransplantation of embryonic day 28 (E28) pig renal primordia into mesentery of nonimmune-suppressed adult pig hosts [6]. However, engraftment and survival of embryonic pig kidney xenografts in immune-competent rodents require that hosts be immunosuppressed [5, 8, 9]. 

**Figure 1:**
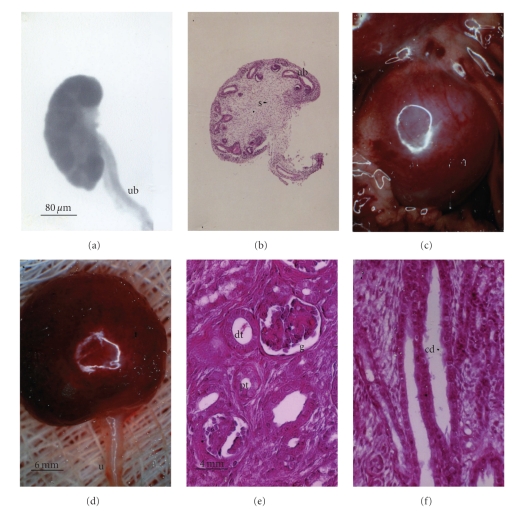
Photographs (a, c, d) and photomicrographs (b, e, f) of E28 pig renal primordia (a, b) or E28 pig renal primordia 7 weeks after transplantation into the mesentery of a rat (c–f). (a) E28 primordium (ub, ureteric bud); (b) E28 primordium (s, stroma; ub, ureteric bud); (c) E28 pig renal primordium 7 weeks after transplantation in a rat mesentery; (d) E28 pig renal primordium after removal from the mesentery (u, ureter), (e) Cortex with a glomerulus, (g) proximal tubule (pt), and distal tubule (dt) labeled; (f) Medulla with collecting duct (cd) labeled. Magnifications are shown for (a) and (b) (in (a)); (c) and (d) (in (d)); (e) and (f) (in (e)) reproduced with permission [8].

We transplanted E28 pig renal primordia (Figures 1(a) and 1(b)) consisting of undifferentiated stroma (s), branched ureteric bud (ub), and primitive developing nephrons into the mesentery of Lewis rats [8, 9] or C57Bl/6J mice [6]. From five to seven weeks after transplantation, no trace of the renal primordium could be found in hosts that received no immunosuppression. In contrast, Figure 1 illustrates undifferentiated E28 pig renal primordia prior to transplantation (Figures 1(a) and 1(b)) and differentiated primordia 6-7 weeks after transplantation into immune-suppressed rats (Figures 1(c), 1(d), 1(e), and 1(f)). The developed pig renal primordium is slightly larger in volume (diameter and weight) than a normal rat kidney [8]. 

Although not tested following transplantation of pig renal primordia into rats, the ultrastructurally normal kidneys that differentiate following allotransplantation of embryonic rat primordia are capable of filtering blood, and urine is excreted following anastomosis between transplant and host ureters. Such transplants support life in otherwise anephric hosts [20, 21]. 

**Figure 2:**
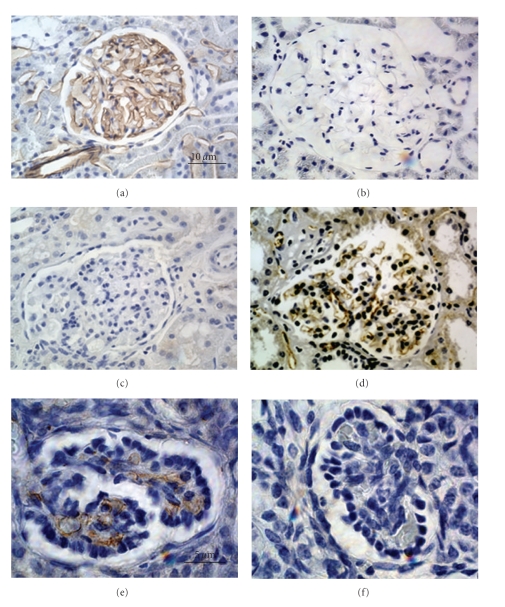
Photomicrographs of stained sections of (a, c) rat kidney; (b, d) pig kidney; (e, f) a pig renal primordium from an E28 embryo 8 weeks after transplantation into a rat mesentery, stained with RECA-1 (a, b, e) or CD31 (c, d, f). Glomerular capillaries in transplants stain positive for RECA-1 that is specific for rat endothelium (e) and negative for CD31 that is specific for pig endothelium (f). Magnifications are shown for (a–d) (in (a)) and (e) and (f) (in (e)), reproduced with permission [9].

Shown in Figure 2 are glomeruli from rat kidneys and pig kidneys and glomeruli within pig renal primordia transplanted into rats 2 weeks previously and stained with antirat endothelial antigen 1 (RECA-1) that is specific for rat endothelium, or anti-CD31 that is specific for pig endothelium. The origin of the glomerular vasculature in transplants is rat (host). Nonglomerular renal vasculature is also of host origin [9]. 

Dekel et al. successfully transplanted renal primordia originating from pig embryos aged E20-21 to E27-28 beneath the renal capsule of immunodeficient mice. Most transplants from the E20–25 donors fail to develop or evolve into growths containing few glomeruli and tubules, but other differentiated derivatives such as blood vessels, cartilage, and bone. In contrast, the transplants originating from E27-28 pig embryos all exhibited significant growth and full differentiation into mature glomeruli and tubule. Dekel et al. found mouse CD31 expression in external vessels as well as developing glomeruli and small capillaries of pig renal primordium xenografts, consistent with a host origin for the vasculature of the developed renal primordium cellular transplants [5]. In addition, Dekel et al. transplanted adult pig kidney tissue or E27-28 pig renal primordia beneath the renal capsule or onto the testicular fat of immunocompetent Balb/c amice. Some hosts were treated with CTLA4-Ig. Evaluation of adult or E27-28 embryonic tissues 2 weeks after implantation into non-CTLA4-Ig-treated hosts showed rejection of tissues. In CTLA4-Ig-treated hosts, most E27-28 renal primordia underwent growth and differentiation. In contrast, all adult kidney grafts had a disturbed morphology, necrotic tissue, and a high degree of lymphocyte infiltration. The authors interpreted these data as being consistent with an immune advantage of the developing precursor transplants over developed adult kidney transplants in fully immunocompetent hosts [5].

Dekel and coworkers implanted metanephroi from E70 human embryos intraperitoneally into immunodeficient (SCID) mice. Transplanted kidneys survived for more than 2 months after transplantation [4]. Hybridization to cDNA arrays of RNA derived from normal human renal primordia at 8, 12, 16, or 20 weeks of gestation demonstrated a subset of 240 genes, the expressions of which changed substantially with time. Clustering analysis of global gene expression in transplants after transplantation revealed a temporal profile of gene expression similar to that observed in the normal human kidneys during development, consistent with recapitulation of a renal developmental program. Comparison of the expression profiles of developing metanephroi to a Wilms' tumor specimen revealed no similarity consistent with no threat of malignant transformation after transplantation of human kidney precursors.

## 3. Xenotransplantation of Embryonic Pig Pancreas

We have shown that glucose tolerance can be normalized in streptozotocin- (STZ-) diabetic (type 1) LEW [7, 8, 12] rats or ZDF (type 2) diabetic rats [10] within 4 weeks following transplantation in mesentery of pig pancreatic primordia obtained very early during embryogenesis (on embryonic day 28 (E28)—just after the organ differentiates and prior to the time dorsal and ventral anlagen fuse) without host immune suppression. Rats are rendered permanently independent of a requirement for exogenous insulin to maintain normoglycemia. No circulating rat insulin can be detected in STZ-treated rats. Porcine insulin circulates after transplantation of E28 pig pancreatic primordia (embryonic pancreas), and levels increase after a glucose load. Cells expressing insulin and porcine proinsulin mRNA with beta cell morphology engraft in host mesentery, mesenteric lymph nodes, liver, and pancreas after transplantation. Cells originating from E28 pig pancreatic primordia engraft similarly in nonimmune-suppressed STZ-diabetic rhesus macaques [11]. 

**Figure 3:**
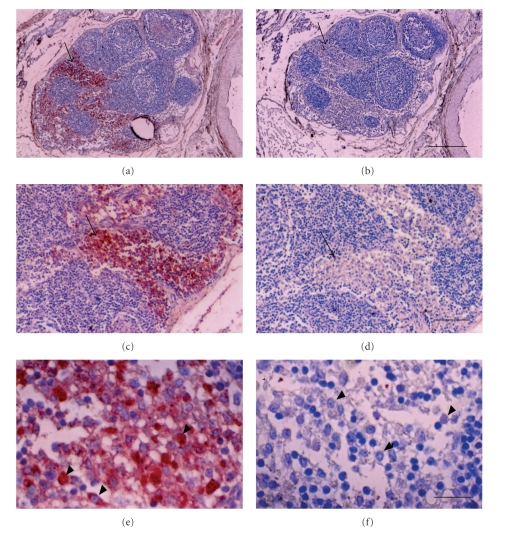
Photomicrographs of mesenteric lymph node from an STZ-diabetic rhesus macaque, 78 days after transplantation of E28 pig pancreatic primordia. Sections (a), (c), and (e) are stained with an anti-insulin antibody. Sections (b), (e), and (f) are stained using a control serum. Sections of medullary sinus are delineated by arrows (a–d). Individual cells with beta cell morphology are delineated by arrowheads (e) and (f). Scale bars 120 um (a) and (b); 80 um (c) and (d); 20 um (e) and (f), reproduced with permission [11].

Figure 3 shows photomicrographs originating from a mesenteric lymph node of a transplanted rhesus macaque. Sections in Figures 3(a), 3(c), and 3(e) are stained with an anti-insulin antibody. Sections in Figures 3(b), 3(d), and 3(f) are incubated with control serum. Sections of medullary sinus are delineated by arrows (Figures 3(a), 3(b), 3(c), and 3(d)). Individual cells with beta cell morphology that stain positive (red) are delineated by arrowheads (Figure 3(e)). No positive-staining cells are found in sections treated with control serum (Figures 3(b), 3(d), and 3(f). Cells with morphology similar to positive cells in Figure 3(e) are delineated in Figure 3(f) (arrowheads). No insulin-positive cells are present in mesenteric lymph nodes of nontransplanted rhesus macaques [11].

**Figure 4:**
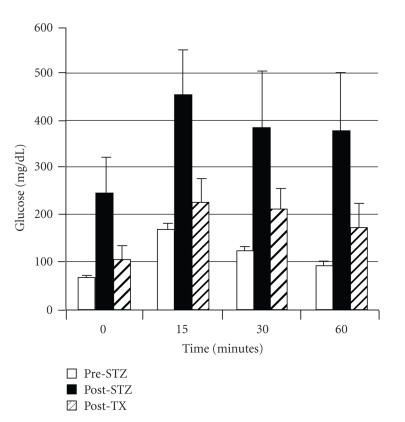
Intravenous glucose tolerance in rhesus macaques. Glucose in peripheral venous blood was measured prior to intravenous infusion of dextrose (Time 0) and at several times after infusion in three fasted rhesus macaques either prior to administration of STZ (Pre-STZ), 5 days following administration of STZ (Post-STZ), or 3 months following transplantation of 20–40 E28 pig pancreatic primordia in mesentery of STZ-diabetic macaques (Post-TX). Data are shown as mean ± SE (3 macaques), reproduced with permission [13].

As shown in Figure 4, glucose tolerance can be nearly normalized in nonimmune-suppressed diabetic rhesus macaques following transplantation of E28 pig pancreatic primordia [13]. Exogenous insulin requirements are reduced in transplanted macaques [11], and animals have been weaned off insulin for short periods of time, but not permanently. The most likely explanation for the difference between rats and macaques is that macaques weigh 20 times as much as rats. An STZ-diabetic rat can be rendered normoglycemic lifelong with no exogenous insulin requirement by transplantation of 5–8 pig pancreatic primordia. Extrapolating, it would take 100–160 primordia to render a diabetic rhesus macaque independent of exogenous insulin. This would require the sacrifice of about 7–12 pregnant sows and multiple surgeries [12] with the attendant complications.

In lieu of increasing the number of transplanted primordia in diabetic rhesus macaques, we embarked on a series of experiments to determine whether porcine islets, a more easily obtainable and possibly more robust source of insulin-producing cells, could be substituted in animals rendered tolerant to embryonic pig pancreas. Our first step was to determine using rats, whether engraftment of cells originating from E28 pig pancreatic primordia renders hosts tolerant to the same or similar cell component present in porcine islets from adult swine (adult islets). To this end, we implanted adult porcine islets beneath the renal capsule of rats that previously had been transplanted with E28 pig pancreatic primordia in mesentery [12].

**Figure 5:**
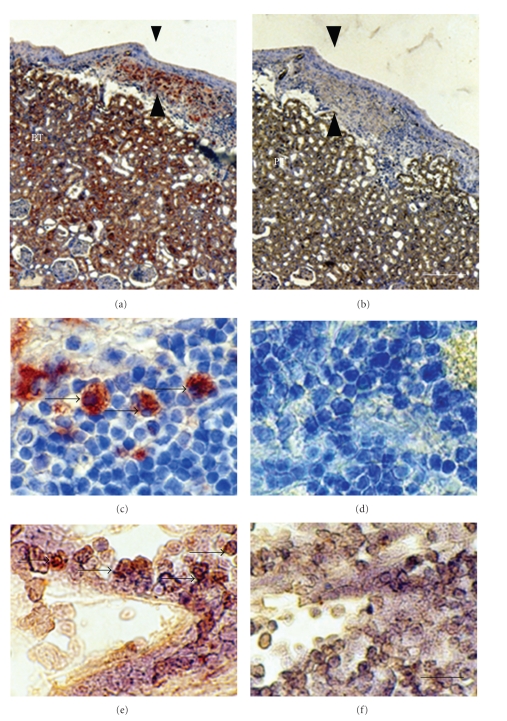
Photomicrographs of kidney from a diabetic rat into which embryonic pig pancreas had been transplanted in mesentery, and pig islets had been transplanted subsequently in kidney stained using anti-insulin antibody (a, c) or control antibody (b, d) and sections hybridized to antisense (e) or sense (f) porcine proinsulin mRNA probes. Arrowheads delineate an expanded subcapsular space (a, b). Arrows delineate tissue in the subcapsular space that stains positive for insulin (red-brown) (c) or positive staining for porcine proinsulin mRNA (e). PT, proximal tubule (a, b). Scale bars 80 um (a, b) and 10 um (c–f), reproduced with permission from the American Society for Investigative Pathology [12].

Figure 5 shows sections from a kidney from an STZ-diabetic rat transplanted previously with embryonic pig pancreas in mesentery and subsequently with pig islets in one kidney. Sections are stained using anti-insulin antibodies (Figures 5(a) and 5(c)) or control serum (Figures 5(b) and 5(d)). As would be expected for kidney that filters, reabsorbs, and secretes insulin, proximal tubules (PTs) in Figure 5(a) are positive (red brown) relative to comparable structures in Figure 5(b). Cells that stain for insulin (Figure 5(a)), but not with control serum (Figure 5(b)) are present in an expanded subcapsular space (Figures 5(a) and 5(b); arrowheads).  Figure 5(c) shows a higher magnification of the subcapsular space. The cells that stain positive for insulin (red-brown stain) are polygonal with round nuclei and abundant cytoplasm (arrow), a beta cell morphology. Also shown in Figure 5 are sections incubated with antisense (Figure 5(e)) or sense (Figure 5(f)) porcine proinsulin mRNA probes. Hybridization occurs following incubation with the former (arrows), but not the latter. In contrast to the transplanted kidney, there is no expansion of the subcapsular space in the contralateral (nontransplanted) kidney and no cells are present with beta cell morphology. The subcapsular space of a kidney from a rat implanted with porcine islets four weeks previously with no prior transplantation of E28 pig pancreatic primordia is expanded relative to that of a nontransplanted kidney. However, there are no cells with beta cell morphology that stain for insulin [12]. 

Schroeder et al. [22] define transplantation tolerance as immune unresponsiveness to the transplanted organ, but not to other antigens, in the absence of ongoing immunosuppression. LEW rats transplanted with E28 pig pancreatic primordia retain reactivity to other porcine xenoantigens (E28 pig renal primordia are rejected) [8]. Thus, our findings are consistent with induction of specific tolerance [22] to a cell component (either beta cells or a stem cell component that differentiates into insulin-producing cells) of adult porcine islets implanted in LEW rats by previous transplantation of E28 pig pancreatic primordia. 

Though not observed following xenotransplantation under all conditions [16, 17], host tolerance to early stage pancreatic progenitors has been reported twice previously. Eloy et al. described normalization of glucose after transplantation of E15, but not E18 embryonic chick pancreas into non-immune-suppressed STZ-diabetic immune-competent rats [23]. Abraham et al. [24] described successful xenoengraftment in multiple organs of human pancreatic islet-derived progenitor cells infused in nonimmunosuppressed immune-competent mice. It is possible that xenotransplantation of fetal pancreas is particularly suited to induction of tolerance. However, neither Eloy et al. [23], nor Abraham et al. [24], nor we [7, 8, 10–12] define an immunological mechanism. 

Although the antigenicity of fetal tissues may be less than that of corresponding adult tissues, animal data suggest the reduction is not enough by itself to ensure permanent graft survival [25]. Thus, the use of embryonic tissue (pancreas) per se cannot explain our findings. Host immune suppression is required for successful engraftment of embryonic pig pancreas in rodents [16] or nonhuman primates [17] carried out using methodology different from ours. Therefore, it is likely that one or more differential factors in the methodology we employ are critical for engraftment of embryonic pig pancreas without an immune suppression requirement. Such factors could include the developmental stage of embryos from which primordia are obtained, the number of pancreatic primordia transplanted, the manner in which embryonic pancreas organs are incubated in vitro prior to implantation, the diabetic status of the host, the transplantation site and methodology for securing the implants in place, and the stringency by which glucose levels in diabetic hosts are controlled after transplantation [13].

We have proposed [13] that transplantation of E28 pig pancreatic primordia in the mesentery and migration of cells to mesenteric lymph nodes and liver recapitulates events that occur during induction of oral tolerance [26–28], the induction of which is dependent on antigen transport via afferent lymphatics into the draining mesenteric lymph nodes [28]. In effect, we suggest that heterotopic introduction of embryonic pig pancreas in rat or primate mesentery co-opts the function of the gut-associated lymphoid tissues (GALT), a complex redundant [26–28] and phylogenetically ancient system [29, 30] of which embryonic pancreas is a part [31], that under normal conditions induces peripheral tolerance to ingested antigens in jawed vertebrates and their descendants. Harada et al. have proposed a similar co-opting of oral tolerance to explain the muted immune response in vivo and by cells from mesenteric lymph nodes in vitro to a colon carcinoma of BALB/c origin or a human CD80-transfected DBA/2 mastocytoma injected into the subserosa of cecum in BALB/c mice relative to tumors injected subcutaneously [32]. GALT may have served similarly to prevent an immune response to insulin-producing cells scattered originally in the gut epithelium of primitive vertebrates [29, 30] and have been proposed to induce tolerance or immune suppression towards islet cell antigens during normal embryonic development [31].

## 4. Perspectives

Dialysis as a therapy for end-stage kidney failure is life preserving but replaces only a small fraction of normal kidney function and has and considerable morbidity. Kidney allotransplantation provides a higher level of renal function and a less constrained lifestyle. However, it is limited by the number of human organs available [33]. 

Use of oral hypoglycemic agents and administration of insulin are cornerstones of treatment for diabetes mellitus. However, adequate control of circulating glucose levels cannot be attained by most patients, and attempts at maintaining euglycemia through intensive insulin therapy lead to hypoglycemia. In contrast, allotransplantation therapies (whole pancreas or islets of Langerhans (islets)) can normalize glucose control [1, 2]. Given existing technology, a major limitation to the use of either whole pancreas or islet allotransplantation is the insufficient supply of human organs. Compounding this limitation for islet transplantation is the need to transplant large quantities of islets from multiple donors to achieve even short-term insulin sufficiency. Selection criteria at most US centers for pancreas transplantation dictate a conservative approach that excludes most type 2 diabetics, traditionally older and poorer surgical risks than type 1 patients.

Whole pancreas transplantation requires use of potent immunosuppressive medications that have significant complications. Newer, more targeted immunosuppressive regimens that do not require steroid or high-dose calcineurin inhibitors make islet transplantation a more attractive option. However, side effects of immune suppression that must be maintained so long as the graft functions remain a source of morbidity and even mortality [34]. Thus, transplantation therapy for diabetes trades one set of morbidities (associated with diabetes and its medical treatment) for another (associated with immune suppression). 

In that pigs are plentiful and have renal function very similar to humans, and because porcine insulin works well in humans, the pig has been suggested as a kidney or pancreas organ donor for humans with chronic kidney disease or diabetes. While humoral rejection is ameliorated following transplantation into nonhuman primates of kidneys from pigs transgenic for the human complement activator, decay accelerating factor [35], or the use of kidneys or organs from transgenics that do not express alpha 1,3 galactosyltransferase [36], neither the immunosuppressive regimens used for pig to primate kidney transplantation nor the outcomes would be acceptable in humans. The severity of humoral rejection effectively precludes the use of pigs as whole pancreas organ donors for humans. However, because they are vascularized by the host after transplantation, islets like other cell transplants are not subject to humoral rejection. Recent experience with pig to primate islet or neonatal islet transplantation shows that sustained insulin independence can be achieved, but only through the use of immunosuppressive agents that are not approved for human use or that result in a high level of morbidity and mortality in diabetic primates [37–39]. Thus, the need for host immune suppression is a barrier for pig-to-primate kidney or islet xenotransplantation. 

The use of individual cells including stem cells to regenerate or repair damaged kidney tissue or differentiate into insulin-producing cells (cell therapies) offers an alternative to whole organ replacement. Since cells are nonvascularized, these approaches can circumvent humoral rejection of xenogeneic tissue mediated by preformed antibodies directed against donor endothelial antigens. However, cell transplantation to replace the function of a structurally complex organs such as the kidney has limitations. In order for glomerular filtration, reabsorption, and secretion of fluid and electrolytes to take place in a manner that will sustain life, individual nephrons must be integrated in three dimensions with one another and with a collecting system, the origin of which is yet another separate structure, the ureteric bud. Concomitantly, vascularization must occur in a unique organ-specific manner from endothelial precursors that may originate from both inside and outside of the developing renal primordium. While it is conceivable that endocrine functions of the kidney, such as erythropoietin production, could be recapitulated by transplanting one particular type of renal cell and it is possible that replacement of one or another type of injured renal cell could enhance the function of damaged tubules, it is difficult to imagine how glomerular filtration and reabsorption in kidneys could be reconstituted de novo by infusion of individual cells [33]. Recent studies have generated pancreatic cells from human ES cells. However, successful differentiation in vitro of functional beta cells remains an elusive goal [40]. 

Xenotransplantation of embryonic pig renal or pancreatic primordia in lieu of mature pig organs or porcine islets couples the wide availability of whole pig organs with the immunological advantages inherent in transplanting cellular embryonic tissue, circumventing humoral rejection, and in the case of pancreas, obviating the need for host immune suppression. Furthermore, unlike ES cells, renal and pancreatic primordia are programmed to differentiate into anatomically precise kidneys or beta cells in which glucose sensing and insulin release are functionally linked. Finally, prior transplantation of pig pancreatic primordia permits the engraftment of an insulin-producing cell component originating from porcine islets implanted subsequently. 

Xenotransplantation of embryonic pig kidney or pancreas, once employed safely and effectively in humans, will provide in essence an unlimited supply of donor organs. This will result in a paradigm shift in how the world thinks about organ replacement: (1) there will be no need to transport organs across long distances; (2) transplantation can be done electively at a convenient time; (3) transplantation can be offered to high-risk individuals and can be repeated as needed; (4) transplantation can be offered to patients currently not candidates including type 2 diabetics.
